# Supplementary material for: An analysis of abnormalities in the B cell receptor repertoire in patients with systemic sclerosis using high-throughput sequencing
Source: PeerJ. 2020 Jan 14;8:e8370. doi: 10.7717/peerj.8370 (PMC6968515; doi:10.7717/peerj.8370)
Supplement: Table S1 [file peerj-08-8370-s001.doc]

**Table S1.** Multiplex PCR primers for the amplification of the IGH CDR3 region.

| Primers | Sequences |
| --- | --- |
| IGHV1-18 | CAGACGTGTGCTCTTCCGATCTAGAGAGTCACCATGACCACAGAC |
| IGHV1-2/1-46 | CAGACGTGTGCTCTTCCGATCTAGAGAGTCACCAKKACCAGGGAC |
| IGHV1-24 | CAGACGTGTGCTCTTCCGATCTAGAGAGTCACCATGACCGAGGAC |
| IGHV1-3/1-45 | CAGACGTGTGCTCTTCCGATCTAGAGAGTCACCATTACYAGGGAC |
| IGHV1-69/1-f | CAGACGTGTGCTCTTCCGATCTAGAGAGTCACGATWACCRCGGAC |
| IGHV1-8 | CAGACGTGTGCTCTTCCGATCTAGAGAGTCACCATGACCAGGAAC |
| IGH2-70/26/5 | CAGACGTGTGCTCTTCCGATCTAGACCAGGCTCACCATYWCCAAGG |
| IGHV3 | CAGACGTGTGCTCTTCCGATCTAGGGCCGATTCACCATCTCMAG |
| IGH4 | CAGACGTGTGCTCTTCCGATCTAGCGAGTCACCATRTCMGTAGAC |
| IGHV5-51 | CAGACGTGTGCTCTTCCGATCTAGCAGCCGACAAGTCCATCAGC |
| IGHV6-1 | CAGACGTGTGCTCTTCCGATCTAGAGTCGAATAACCATCAACCCAG |
| IGHV7-NEW | CAGACGTGTGCTCTTCCGATCTAGGACGGTTTGTCTTCTCCTTG |
| HIGHJ-Rev1 | CTACACGACGCTCTTCCGATCTCTGAGGAGACRGTGACCAGGGTG |
| HIGHJ-Rev2 | CTACACGACGCTCTTCCGATCTCTGAAGAGACGGTGACCATTGTC |
| HIGHJ-Rev3 | CTACACGACGCTCTTCCGATCTCTGAGGAGACGGTGACCAGGGT |
| HIGHJ-Rev4 | CTACACGACGCTCTTCCGATCTTGAGGAGACGGTGACCGTGGTC |
