# Supplementary material for: An analysis of abnormalities in the B cell receptor repertoire in patients with systemic sclerosis using high-throughput sequencing
Source: PeerJ. 2020 Jan 14;8:e8370. doi: 10.7717/peerj.8370 (PMC6968515; doi:10.7717/peerj.8370)
Supplement: Table S2 [file peerj-08-8370-s002.doc]

| **Table S 2**. Degree of expansion and impact of B cell clones. | | | | | | | | | | |
| --- | --- | --- | --- | --- | --- | --- | --- | --- | --- | --- |
| Degree of expansion | | Clones (%) | | | | | | | | P |
|  |  | SSc (Mean ) | | SSc (SD) | | Control (Mean ) | | Control (SD) | |  |
| Rare (<0.0005%) | 0.874 | | 3.32×10^-2^ | | 0.909 | | 3.72×10^-3^ | | 0.019 | |
| Low (0.0005-<0.005%) | 1.95×0^-2^ | | 9.71×10^-3^ | | 1.25×10^-2^ | | 1.37×10^-3^ | | 0.084 | |
| Medium (0.005-<0.05%) | 9.37×10^-2^ | | 3.51×10^-2^ | | 4.3×10^-2^ | | 1.24×10^-2^ | | 0.005 | |
| High (0.05-<0.5%) | 1.31×10^-2^ | | 1.42×10^-2^ | | 3.51×10^-2^ | | 9.9×10^-3^ | | 0.013 | |
| Expanded (≥0.5%) | | 4.76×10^-6^ | | 1.35×10^-5^ | | 9.85×10^-6^ | | 1.97×10^-5^ | | 0.664 |
