# Supplementary figures and images for: An analysis of abnormalities in the B cell receptor repertoire in patients with systemic sclerosis using high-throughput sequencing
Source: PeerJ. 2020 Jan 14;8:e8370. doi: 10.7717/peerj.8370 (PMC6968515; doi:10.7717/peerj.8370)

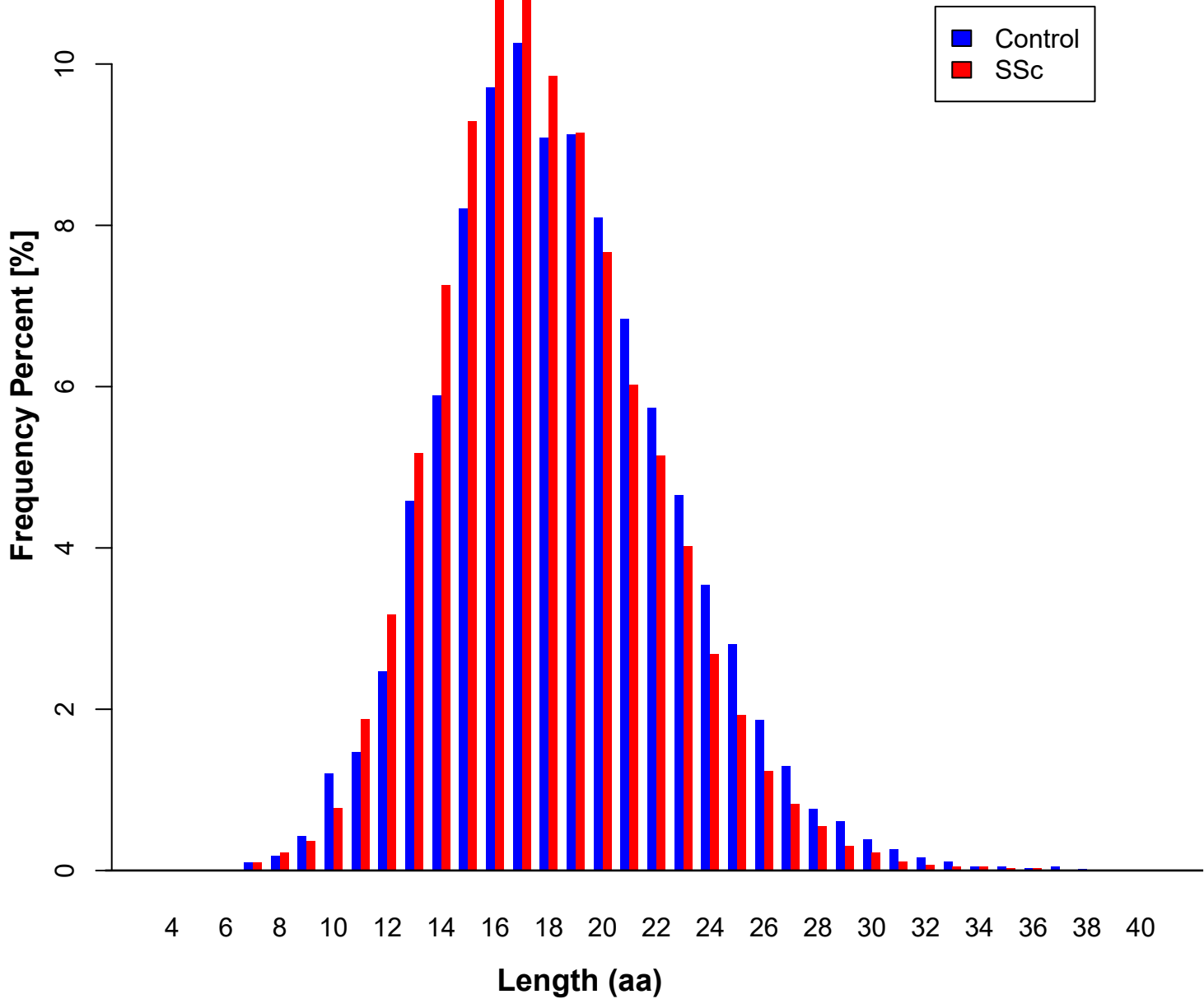

Supplement: Figure S1 [file peerj-08-8370-s003.pdf]

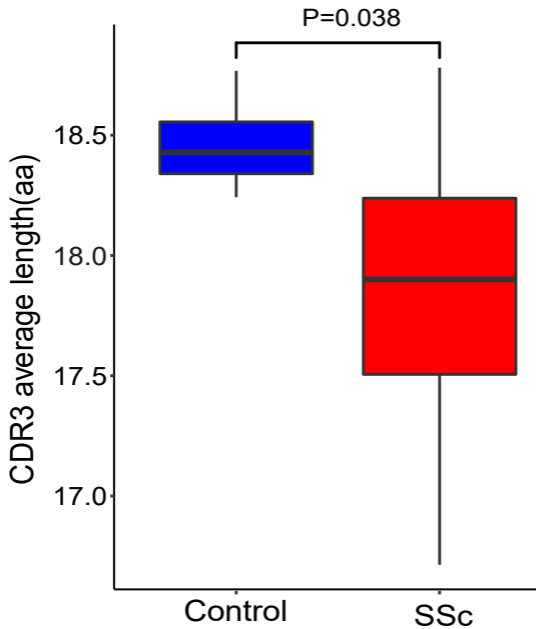

Supplement: Figure S2 [file peerj-08-8370-s004.pdf]

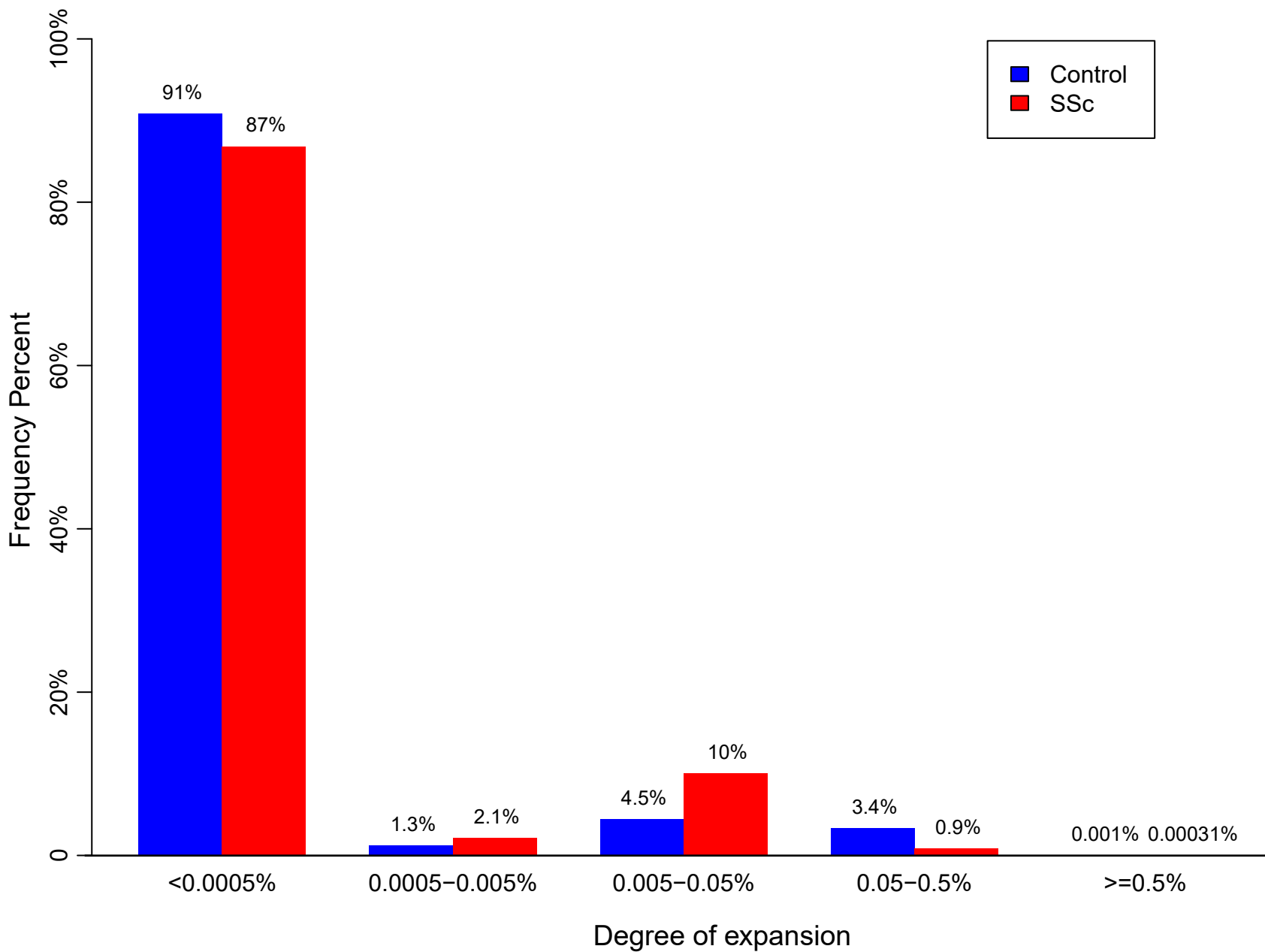

Supplement: Figure S3 [file peerj-08-8370-s005.pdf]
